# COVID-19 and Influenza Vaccination Campaign in a Research and University Hospital in Milan, Italy

**DOI:** 10.3390/ijerph19116500

**Published:** 2022-05-26

**Authors:** Maurizio Lecce, Giacomo Biganzoli, Luca Agnello, Ignazio Belisario, Giovanni Cicconi, Marilena D’Amico, Francesca De Giorgi, Angelo Ferilli, Gaia Ferraguzzi, Fabio Guzzardi, Danilo Lanzillotti, Roberta Lattanzio, Chiara Marrocu, Maria Emanuela Noto, Sara Piccinelli, Noemi Sabatelli, Sheila Santisteban, Sudwaric Sharma, Livia Tognaccini, Silvana Castaldi

**Affiliations:** 1 Department of Biomedical Sciences for Health, Postgraduate School of Public Health, University of Milan, 20136 Milan, Italy; luca.agnello1@unimi.it (L.A.); ignazio.belisario@unimi.it (I.B.); giovanni.cicconi@unimi.it (G.C.); marilena.damico@unimi.it (M.D.); francesca.degiorgi1@unimi.it (F.D.G.); angelo.ferilli@unimi.it (A.F.); gaia.ferraguzzi@unimi.it (G.F.); fabio.guzzardi@unimi.it (F.G.); danilo.lanzillotti@unimi.it (D.L.); roberta.lattanzio@unimi.it (R.L.); chiara.marrocu@unimi.it (C.M.); mariaemanuela.noto@unimi.it (M.E.N.); sara.piccinelli@unimi.it (S.P.); noemi.sabatelli@unimi.it (N.S.); sheila.santisteban@unimi.it (S.S.); sudwaric.sharma@unimi.it (S.S.); livia.tognaccini@unimi.it (L.T.); silvana.castaldi@unimi.it (S.C.); 2 Unit of Medical Statistics, Biometry and Epidemiology, Department of Biomedical and Clinical Sciences (DIBIC) “L. Sacco” & DSRC, Luigi Sacco University Hospital, 20157 Milan, Italy; giaco.biganzoli@gmail.com; 3 Fondazione IRCCS Ca’ Granda Ospedale Maggiore Policlinico, 20122 Milan, Italy

**Keywords:** influenza vaccine, healthcare workers, COVID-19 vaccine, vaccine coadministration, vaccination coverage, vaccine acceptance, vaccine adherence, vaccine compliance

## Abstract

Background: Healthcare workers (HCWs) are a historical key target of influenza vaccination programs. For the 2021–2022 season, WHO considered the coadministration of a flu and a COVID-19 vaccine as acceptable and recommended it to allow for higher uptake of both vaccines. The aim of this study was to investigate demographic and occupational features of vaccinated HCWs, reasons behind flu vaccine acceptance and a possible effect of the coadministration of a COVID-19 vaccine, in order to potentially draw general conclusions on HCWs’ attitude towards flu vaccination and inform further strategies for consistent improvement of vaccine acceptance. Methods: a promotional and educational campaign, a gaming strategy, and vaccination delivery through both a large central hub and on-site ambulatories, were the implemented strategies. In the central hub, the flu/COVID-19 vaccine coadministration was offered. Statistical descriptive analysis, multiple correspondence analysis (MCA) and logistic regression models were performed. Results: 2381 HCWs received the flu vaccine, prompting a vaccination coverage rate (VCR) of 52.0% versus 43.1% in the 2020–2021 campaign. Furthermore, 50.6% vaccinated HCWs belonged to the 18–39 years-old age group. The most expressed reasons for vaccine uptake were “Vaccination is the most effective strategy of prevention” (n = 1928, 81.0%), “As HCW it’s my duty to get vaccinated to protect my patients” (n = 766, 32.2%), and the group of COVID-19-related reasons (n = 586, 24.6%). In addition, 23.3% HCWs received the flu vaccine in the current campaign but not in the previous one (newly vaccinated) and the flu/COVID-19 vaccine coadministration was more frequent in this group. A total of 51.0% HCWs were hesitant towards the coadministration, while residents and nurses showed the highest propensity to receive it. Conclusions: in the second year of the COVID-19 pandemic, the Fondazione’s influenza VCR continued to increase, with the greatest participation among HCWs aged 18–39 years. A potential propelling role of the COVID-19 vaccine coadministration was highlighted.

## 1. Introduction

Influenza is an acute respiratory infection caused by influenza A, B, and C viruses, which occurs in local outbreaks or seasonal epidemics [1]. The global annual number of influenza-associated respiratory deaths was estimated to be 290,000–650,000 [2]. The estimate does not take into account deaths from other diseases, e.g., cardiovascular diseases, which can be influenza-related [2]. In Europe, seasonal influenza causes 4–50 million symptomatic cases and 15,000–70,000 annual deaths [3].

Annual influenza vaccination is the most effective way to prevent influenza and it is especially important for people at higher risk of serious complications, such as individuals with specific chronic medical conditions, pregnant women, children aged 6–59 months, and the elderly [4]. In Italy, the recommended vaccination coverage rate (VCR) target is 75%, and 95% is the optimal value for populations at risk [5,6].

As healthcare workers (HCWs) are at higher risk of acquiring influenza virus infection and spreading it to vulnerable individuals, they are a key target of influenza vaccination programs [7,8].

Reaching the recommended target is challenging [9,10], with vaccination coverage rates (VCR) in Italy rarely exceeding the threshold of 20%, even in recent years [11,12,13,14], despite the fact that a wide range of strategies have been adopted in order to facilitate vaccine uptake and increase immunization rates [15,16,17].

Vaccine hesitancy is defined as the “delay in acceptance or refusal of vaccination despite availability of vaccination services” and it is influenced by factors such as complacency (low perceived risks of vaccine-preventable diseases and vaccination not deemed a necessary preventive action), convenience (appeal of immunization services, e.g., in terms of easy accessibility), and confidence (the trust in the effectiveness and safety of vaccines, the system that delivers them, and the motivations of policy makers) [18]. Several authors investigated vaccine hesitancy specifically towards influenza vaccination by HCWs, and similarly concluded that it relies on factors such as lack of adequate influenza-specific knowledge, low-risk perception, lack of education on the vaccine efficacy and safety, fears of its potential side effects, and lack of access to vaccination facilities [19,20,21].

In order to address this public health concern, since the 2019–2020 influenza season, the annual vaccination campaign for HCWs in Fondazione IRCCS Ca’ Granda Ospedale Maggiore Policlinico (thereafter Fondazione), a research and teaching hospital in Milan, Italy, was taken up by the Postgraduate School of Public Health of the University of Milan, with the aim to implement innovative strategies and improve influenza immunization rates. In the 2019–2020 campaign, the main strategy consisted of delivering the flu vaccine by several on-site ambulatories scattered throughout the hospital buildings, besides an already-established ad hoc ambulatory, in order to maximize ease of access to vaccination [22]. However, this single strategy failed to achieve the expected results, as a comprehensive VCR of 21.5% was reached [22]. Following the first wave of the SARS-CoV-2 pandemic, which by February 2020 overwhelmed Italian and worldwide health services [23,24], the 2020–2021 flu vaccination campaign implemented three combined strategy, namely a promotional and educational campaign, a gaming strategy, and a logistically improved on-site and ad hoc vaccine delivery [25]. The final VCR was 43.1% and relevant changes in HCWs attitude towards flu vaccination were registered, foremost a dramatic increase in the participation of HCWs aged 40–59 years [25].

Following the release of the first COVID-19 vaccines in early 2021 [26,27], and in view of the forthcoming 2021–2022 flu season, the World Health Organization (WHO) considered that coadministration of an inactivated seasonal influenza vaccine and any dose of a COVID-19 vaccine was acceptable, and available evidence did not show an increase in adverse events [28]. In addition, WHO recommended considering coadministration to allow for more programmatic ease and higher uptake of both vaccines [28]. As a consequence, vaccination programs against influenza in Italy have been implemented in close association with those against COVID-19 [29]. For all these reasons, the possibility of coadministration was offered also to HCWs in Fondazione, and this was the main addition to the 2021–2022 influenza vaccination campaign.

In this new context characterized by the offer of two distinct vaccines, it is noteworthy to consider that HCWs’ decision to get vaccinated with one or the other or both vaccines might be influenced by a COVID-19-related or influenza-related hesitancy, and that these two hesitancies may act independently or be inter-correlated, as shown by other authors [30].

The aim of this study was to assess the level of adherence to flu vaccination by HCWs of Fondazione in the second year of the COVID-19 pandemic, investigating demographic and occupational features of vaccinated HCWs, their reasons behind flu vaccine uptake, the rate and composition of those newly vaccinated, and comparing these results with those of the previous (2020–2021) campaign. Furthermore, we explored a possible influence of the COVID-19 vaccination offer on the flu vaccine uptake. The final rationale of this analysis was to investigate factors associated with HCWs’ intention to get vaccinated against the flu as well as a possible evolution of those factors in very recent years, so as to contribute in understanding the multi-faceted HCWs attitude towards flu vaccination and to suggest consistently tailored interventions to finally reach the recommended immunization target.

## 2. Materials and Methods

The influenza vaccination campaign for HCWs in Fondazione started on the 8 November and was carried out until the 30 November 2021.

Given the encouraging results achieved in the 2020–2021 influenza vaccination campaign, two out of the three strategies adopted in that campaign, namely a promotional and educational campaign and a competition among the hospital departments (so-called gaming strategy), were still adopted for the 2021–2022 campaign [25].

The promotional and educational campaign was conducted using the hospital intranet platform and started in October 2021. In the intranet page dedicated to the campaign, the promotional section consisted of detailed information on times and places to easily access vaccination as well as several claims highlighting vaccination benefits and reasons to get vaccinated (e.g., “Influenza vaccination decreases chances to develop flu-like symptoms, thus to be considered a suspected COVID-19 case, requiring repeated SARS-CoV-2 testing and temporary isolation”; “Get your flu shot to protect your patients and colleagues as well as your family”, etc.). The educational section consisted of addressing and debunking specific misconceptions on influenza or the flu vaccine emerged from a questionnaire administered to HCWs refusing vaccination in the 2019–2020 campaign in Fondazione (e.g., “I am young so I will not catch the flu”, “Flu is not a severe disease”, “I am afraid of the vaccine side effects”, etc.) [22].

The gaming strategy consisted in assigning every HCW to one of the eight hospital departments then triggering a challenge between departments with a formal recognition of the winner department at the end of the campaign. An updated rank showing progressive VCR of every department was posted day-by-day on the campaign intranet page, stimulating HCWs to make their own department win, thus virtuously increasing the comprehensive hospital VCR.

Regarding the third strategy adopted in the 2020–2021 campaign, namely delivering the flu vaccine both through an ad hoc ambulatory and several on-site ambulatories located next to hospital wards, this had to be partially revised. The reasons linked to this decision were primarily the availability of a new large space located at Ponti pavilion, which became the central hub of the vaccination campaign, and a greater number of vaccinating personnel (28 vaccinating doctors, residents of the Postgraduate School of Public Health at the University of Milan). This allowed a reorganization of the spaces and the possibility of constantly keeping several vaccination units active, in a variable number from 7 to 14 depending on the times and the flow expected from the reservations, therefore avoiding long waiting queues. Most of the vaccination lines were located in the central hub, while the remaining lines were located in four peripheral on-site ambulatories. The central hub was equipped with the necessary infrastructures to be able to store and administer the COVID-19 vaccine, also allowing the coadministration of the influenza vaccine. On the other hand, the satellite on-site ambulatories, located in various strategic points of the hospital, only had the possibility to administer the influenza vaccine. The central hub was characterized by long opening times. It was open for 17 days from 8 a.m. to 5:30 p.m. in order to cover two work shifts and to intercept as many HCWs as possible. The peripheral on-site ambulatories had reduced opening hours and initially had a scheduled period of activity of approximately 10 days, subject to bookings. However, the peripheral ambulatories closed after a few days due to a decrease in user flow, making Ponti pavilion the only active site. This may be related to the inability to offer coadministration in the peripheral ambulatories.

A team of two vaccinators was constantly present for each vaccination unit: one was involved in the patient anamnesis and digital uploading of the data in order to complete the correct registration, the other was responsible for the administration of the vaccine. Access to each vaccination site was by appointment in order to avoid personnel queues. Each HCW could book his slot via the hospital intranet platform.

Before getting the flu shot, HCWs had to fill out an anonymous and self-administered questionnaire in order to collect core data for the study analysis. The questionnaire was developed by the working group and was made up of four sections. In the first section, demographic (gender, age) and occupational (qualification, area of activity) features were collected. Concerning qualification, note that the “Other” subgroup included a variety of heterogeneous professionals not elsewhere assignable, such as biologists, pharmacists, psychologists, engineers, physicists, etc. Concerning the area of activity, to allow for a better stratification of vaccinated HCWs, two new areas were introduced in the last (2021–2022) campaign, i.e., Gynecology–Obstetrics and Services, which included Laboratory services, Radiology, Pathology services, Legal Medicine, Occupational Medicine, and Hygiene services. We believe that demographic and occupational features were indispensable information to characterize the population of vaccinated HCWs and to understand the attitude towards flu vaccination of different HCWs subgroups. In the second section, HCWs had to declare a maximum of 2 reasons for their decision to get the flu vaccine. We believe this section was fundamental to understand the main drivers for flu vaccine adherence and to tailor future campaigns that would use those drivers as a leverage to consistently improve HCWs compliance to vaccination. Reasons were imported from a similar questionnaire administered to HCWs undergoing vaccination in the 2020–2021 flu campaign. Imported reasons were: “Vaccination is the most effective strategy of prevention”; “As HCW I am more exposed to the flu”; “I fear the complications of the flu”; “I suffer from diseases at risk of complications”; “I live with fragile patients”; and “As HCW it’s my duty to get vaccinated to protect my patients” [25]. As can be noticed, reasons explored different dimensions behind flu vaccine acceptance which have emerged in the scientific literature [9,21], e.g., the alignment to programmatic recommendations by major health authorities (“Vaccination is the most effective strategy of prevention”), more “egoistic” motivations (“As HCW I am more exposed to the flu”; “I fear the complications of the flu”), even related to a possible HCW’s fragile health status (“I suffer from diseases at risk of complications”), or more “altruistic” motivations, either towards one’s households (“I live with fragile patients”) or towards patients (“As HCW it’s my duty to get vaccinated to protect my patients”). In order to a achieve a better comprehension of HCWs’ motivations, and particularly to explore a potential role of the COVID-19 pandemic in fostering flu vaccine uptake, we added three COVID-19-related reasons, namely “COVID-19 pandemic made me aware that vaccination is useful for myself”; “COVID-19 pandemic made me aware that vaccination is an act of responsibility towards the community”; and “COVID-19 pandemic made me aware of the danger of respiratory infections”. In the third section, HCWs’ influenza vaccination history was explored, as the following question was asked: “Did you get vaccinated against flu last year (2020–2021)?” We believe this point would be helpful in assessing the newly vaccinated rate for the 2021–2022 campaign and to characterize which HCWs subgroups showed a renewed interest towards flu vaccination, as they got vaccinated in this campaign but not in the previous one. In the fourth section, HCWs were asked if they were undergoing both the influenza and the COVID-19 vaccination by coadministration, allowing us to characterize HCWs fully compliant towards WHO recommendations on influenza and COVID-19 co-vaccination [28].

All statistical analyses were conducted using R software, version 4.0.0. The frequencies and proportions for categorical variables were calculated and reported to numerically summarize the distribution of vaccinated HCWs population. Mosaic plots were produced to graphically explore and analyze the pattern of responses related to the reasons for influenza vaccination uptake among all the HCWs’ categories. As a univariate correlation analysis does not account for the overall association structure, we conducted an exploratory multivariate analysis by means of multiple correspondence analysis (MCA), in order to visually understand whether there were specific categories of HCWs associated with particular reasons for vaccination uptake. The results were shown in four plots projecting the distribution of the different qualifications, areas of activity, and reasons for vaccination in the multivariate space defined by the different dimensions computed by the MCA. Further mosaic plots were produced to graphically explore the relationship between HCWs’ previous vaccination status and the categorical variables coding for: (i) HCWs’ area of activity, (ii) HCWs’ qualification, and (iii) HCWs’ decision to get the influenza vaccine and the COVID-19 vaccine the same day by coadministration. Two distinct univariate logistic regression models considering the previous vaccination status as the response variable and the variables coding for the qualification and the area of activity as predictors, respectively, were used to explore possible associations between the different HCWs’ categories and their previous vaccination status. The strength of the associations was studied by computing the odds ratios (OR) with their 95% confidence intervals, represented in two distinct forest plots. The likelihood ratio test was used to assess the overall association between the response and the predictor variable. Two further univariate logistic models were considered to explore the association between influenza and the COVID-19 vaccine coadministration as response variable and HCWs’ qualification and area of activity as predictor variables. Again, the likelihood ratio test was used to verify the overall association between the response and the predictor variable.

No ethical approval was required for this study, according to Regional Law n. 3 year 2012 of the Lombardy Region.

## 3. Results

### 3.1. General Analysis

A total of 2381 HCWs were vaccinated against influenza. Table 1 shows the characteristics of vaccinated HCWs, as well as the VCR of Fondazione’s internal personnel. Data are shown in comparison with the 2020–2021 and 2019–2020 influenza vaccination campaigns.

Out of 2381 vaccinated HCWs, 1795 (75.4%) were directly employed by Fondazione, i.e., the internal personnel, and 586 (24.6%) were students, residents and workers from outsourcing firms. As the overall Fondazione’s internal personnel is made up of a total of 3451 HCWs, a VCR of 52.0% was reached for the internal personnel of Fondazione, compared to 43.1% in 2020–2021 and 21.5% in 2019–2020, showing a consistent increase across the three vaccination campaigns. The female-to-male ratio was approximately 2:1 and did not differ significantly from previous seasons. Interestingly, an inversion of the age trend was observed, as the median age was 36 (interquartile range 25) in the 2019–2020 campaign, then raised to 43 (interquartile range 23) in the 2020–2021 campaign, and finally decreased to 39 (interquartile range 23) in the current campaign. This trend is consistent with major changes across time in the numerosity of the three age groups, i.e., 18–39, 40–59, and ≥60 years old; in 2019–2020, the 18–39 years old group was the most represented (n = 642, 55.7%), while in 2020–2021 a steep increase occurred among HCWs aged 40–59 years (from n = 374 to n = 925) yielding an increase in the median age; finally, in the current campaign, a meaningful raise was registered in the 18–39 years old group (from n = 938 to n = 1204), causing the median age to decrease again. Indeed, in the present campaign, HCWs aged 18–39 years were by far the most represented (n = 1204, 50.6%), compared to HCWs aged 40–59 years (n = 916, 38.5%) and ≥60 years (n = 261, 11.0%). Concerning the qualification, physicians continued to be the most represented professionals in the vaccinated population (n = 603, 25.3%). Residents nearly doubled compared to the previous campaign (from n = 219 to n = 416) and represented the second largest group (17.5%). Nurses immediately followed as the third largest group (n = 414, 17.4%) and remained stable compared to the previous campaign. A meaningful increase was shown by the “Other” group. Students registered a slight decrease (from n = 158 to n = 83), while the remaining groups, i.e., healthcare technicians, auxiliary staff, and administrative staff showed a stable trend. Concerning the areas of activity, the Specialized Medicine and the Services area were by far the most represented, with 554 (23.3%) and 468 (19.7%) vaccinated HCWs, respectively. A comparison with the two previous campaigns is less feasible due to the introduction, as mentioned in Section 2, of two new areas of activity in the last campaign, i.e., Gynecologists–Obstetrics and Services, in favor of a more accurate stratification of vaccinated HCWs. As a result, the Specialized Medicine area, although being the most numerous one, apparently showed an important decrease in vaccinated subjects; however, at least a good amount of these “lost” subjects were simply registered as belonging to the two new areas. Concerning the remaining areas, a comparison is feasible as the General Medicine showed a substantial stability; the Pediatric, Newborn, and Administrative areas registered a slight decrease in vaccinated subjects, and a greater decrease was shown by the Specialized Surgery. On the other hand, the Intensive Care Unit (ICU) and Emergency area and the General Surgery registered a slight increase.

### 3.2. Analysis of Reasons for Influenza Vaccination

Table 2 summarizes the reasons for influenza vaccination expressed by vaccinated HCWs, stratified for the main categories of interest.

The most expressed reasons were reason 1, i.e., “Vaccination is the most effective strategy of prevention” (n = 1928, 81.0%), reason 9, i.e., “As HCW it’s my duty to get vaccinated to protect my patients” (n = 766, 32.2%), reason 5, i.e., “As HCW I am more exposed to the flu” (373, 15.7%), and reason 3, i.e., “COVID-19 pandemic made me aware that vaccination is an act of responsibility towards the community” (n = 330, 13.9%). Interestingly, the COVID-19-related reasons, i.e., reason 2 (“COVID-19 pandemic made me aware that vaccination is useful for myself”), reason 3, and reason 4 (“COVID-19 pandemic made me aware of the danger of respiratory infections”), can be collapsed into a single group which was expressed by 586, or 24.6%, of HCWs, becoming the third most expressed group of reasons following reason 1 and reason 9. Concerning age, HCWs expressing reason 1, which was the most popular, expectedly showed a median age overlapping that of the general population (39, IQR 23). Notably, median ages of HCWs expressing reason 2 (44, IQR 22) and reason 4 (46, IQR 23.5), which are COVID-19 related reasons, and even more greatly reason 6, i.e., “I fear the complications of the flu” (47, IQR 22), reason 7, i.e., “I suffer from diseases at risk of complications” (53.5, IQR 13), and reason 8, i.e., “I live with fragile patients” (45.5, IQR 21), are consistently higher than that of the general vaccinated population. On the contrary, median ages of HCWs expressing reason 5 (36, IQR 22) and reason 9 (33, IQR 18) are interestingly lower than that of the general vaccinated population.

**Figure 1:**
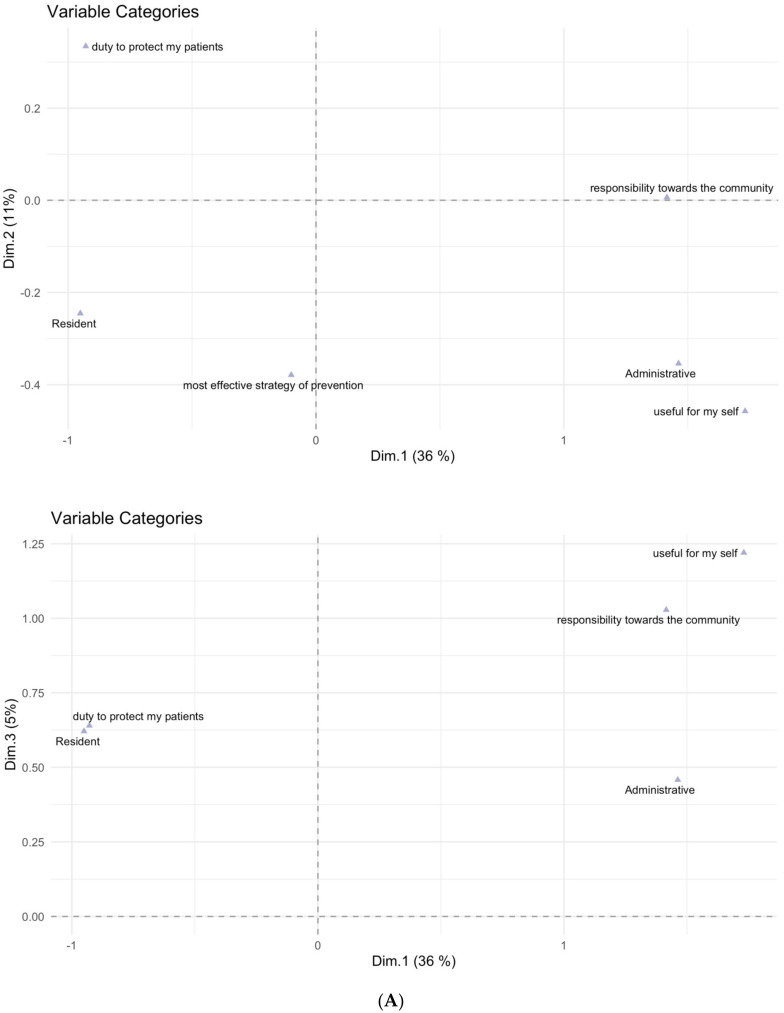

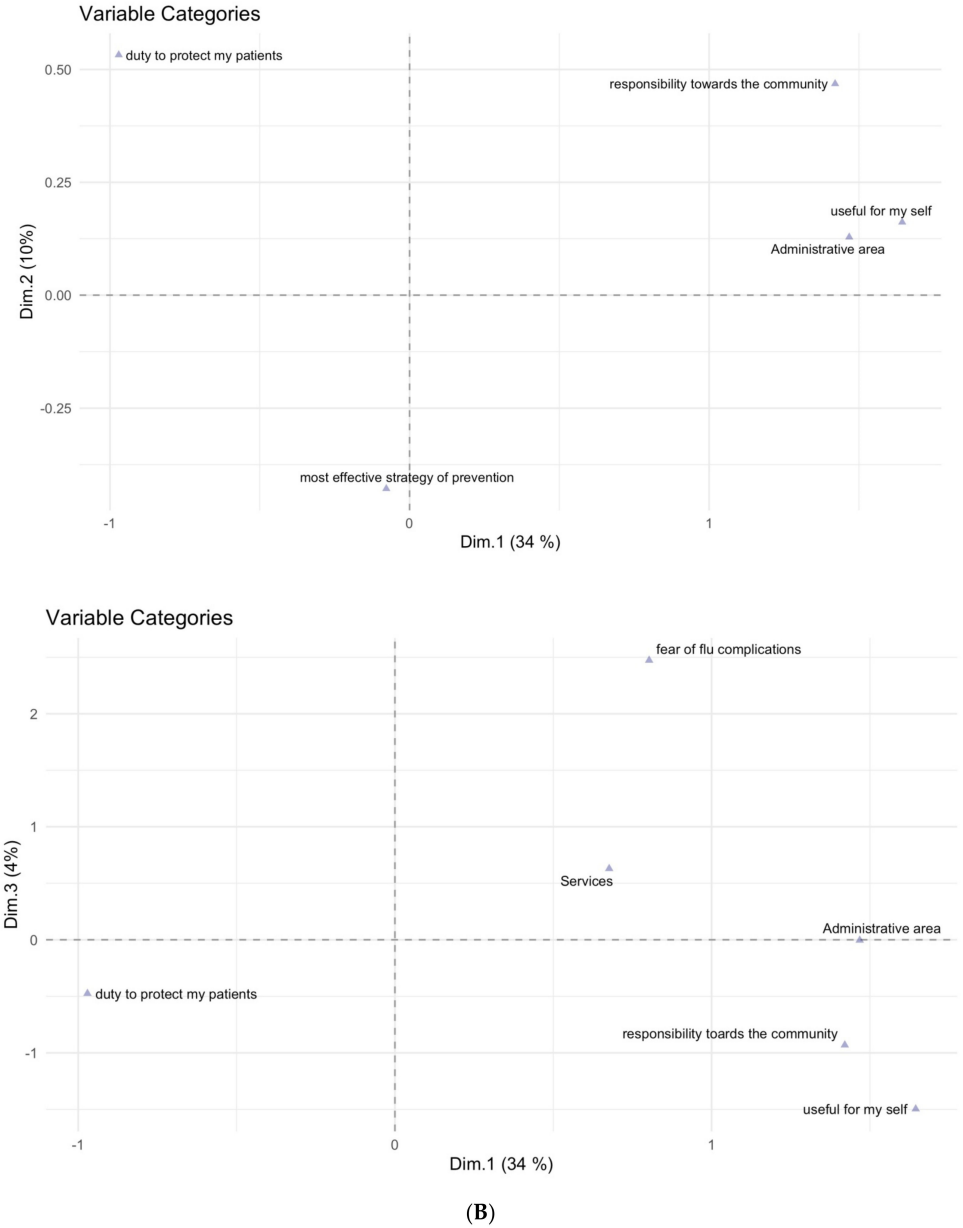
(**A**). HCWs’ qualifications and reasons for flu vaccination uptake resulted to be associated in the new planes defined by the first three dimensions (Dim 1, Dim 2, and Dim 3) computed by the MCA. The three dimensions can be considered as new variables that try to summarize the variability present in the dataset in a quantitative way. This way, particular response profiles characterized by different values of these new dimensions can be extracted from the individual responses that were given to the questionnaire questions. For better interpretability, labels of different reasons have been shortened as follows: reason 1, i.e., “Vaccination is the most effective strategy of prevention” to “most effective strategy of prevention”; reason 2, i.e., “COVID-19 pandemic made me aware that vaccination is useful for myself” to “useful for myself”; reason 3, i.e., “COVID-19 pandemic made me aware that vaccination is an act of responsibility towards the community” to “responsibility towards the community”; and reason 9, i.e., “As HCW it’s my duty to get vaccinated to protect my patients” to “duty to protect my patients”. (**B**). HCWs’ areas of activity and reasons for flu vaccination uptake resulted to be associated in the new planes defined by the first three dimensions (Dim 1, Dim 2, and Dim 3) computed by the MCA. For better interpretability, labels of different reasons have been shortened as follows: reason 1, i.e., “Vaccination is the most effective strategy of prevention” to “most effective strategy of prevention”; reason 2, i.e., “COVID-19 pandemic made me aware that vaccination is useful for myself” to “useful for myself”; reason 3, i.e., “COVID-19 pandemic made me aware that vaccination is an act of responsibility towards the community” to “responsibility towards the community”; reason 6, i.e., “I fear the complications of the flu” to “fear of flu complications”; and reason 9, i.e., “As HCW it’s my duty to get vaccinated to protect my patients” to “duty to protect my patients”.

Concerning qualification and area of activity, an explorative analysis conducted by means of multiple correspondence analysis (MCA) was performed, leading to results shown in Figure 1A,B.

As can be observed in Figure 1A, the administrative personnel is shown to be associated with reason 2 (“COVID-19 pandemic made me aware that vaccination is useful for myself”) and reason 3 (“COVID-19 pandemic made me aware that vaccination is an act of responsibility towards the community”), while residents are strongly associated with reason 9 (“As HCW it’s my duty to get vaccinated to protect my patients”). Figure 1B similarly shows that the administrative area is associated with reason 2 and reason 3.

### 3.3. Newly Vaccinated Analysis

Table 3 shows the influenza vaccination history of vaccinated HCWs in the 2021–2022 campaign as well as for the 2020–2021 and 2019–2020 campaigns.

In the current campaign, 1834 (76.7%) vaccinated HCWs stated they also received the flu vaccine in the previous season (2020–2021), while 547 (23.3%) did not, being newly vaccinated. Data from the 2020–2021 and 2019–2020 campaigns are slightly different, as then HCWs were asked if they ever got vaccinated against flu in the past (not specifically in the previous season). However, 845 out of 2103 (40.2%) vaccinated HCWs in the 2020–2021 campaign and 376 out of 1153 (32.6%) in the 2019–2020 campaign were registered as “never vaccinated before”, i.e., newly vaccinated. Interestingly, the newly vaccinated rate increased from 2019–2020 to 2020–2021 campaign (from 32.6% to 40.2%), then decreased in the current season (from 40.2% to 23.3%).

**Figure 2:**
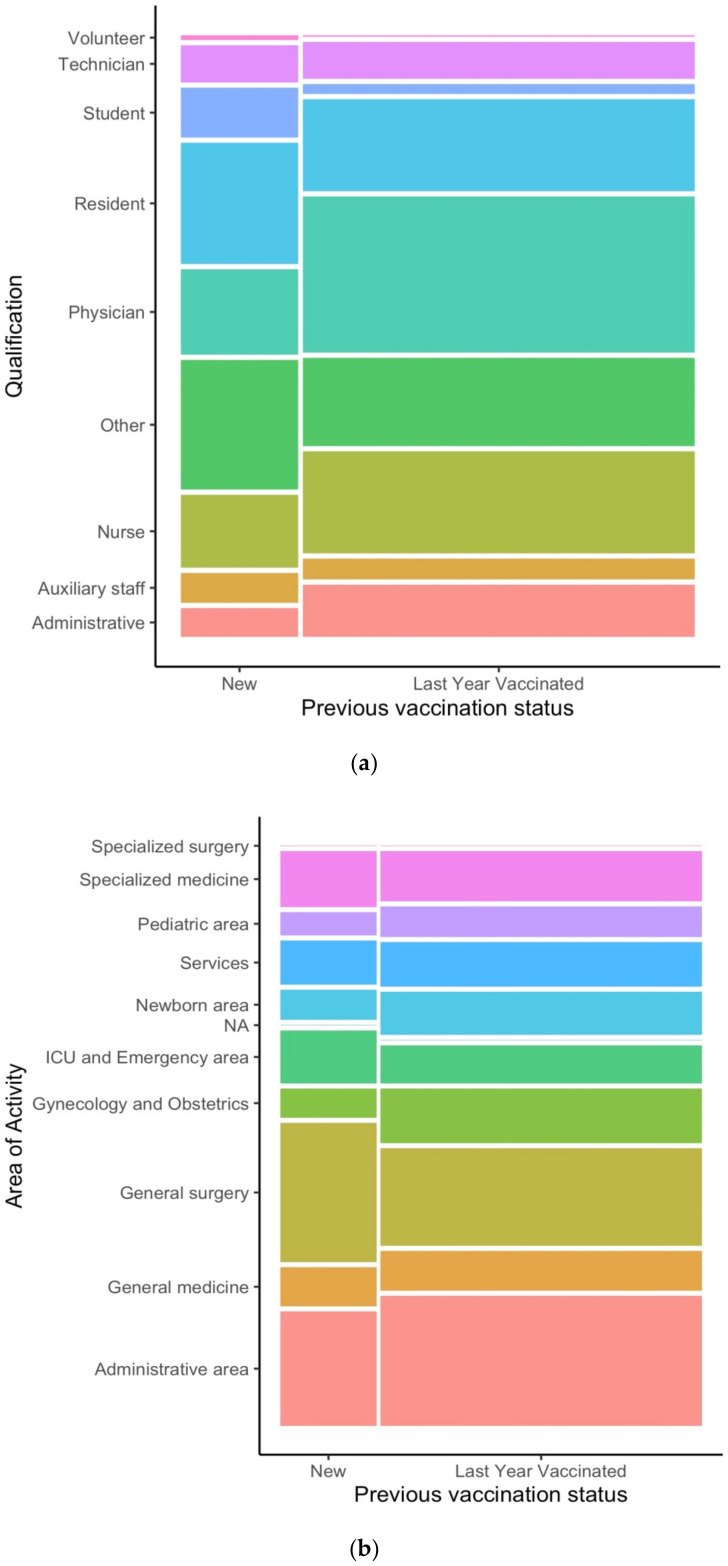

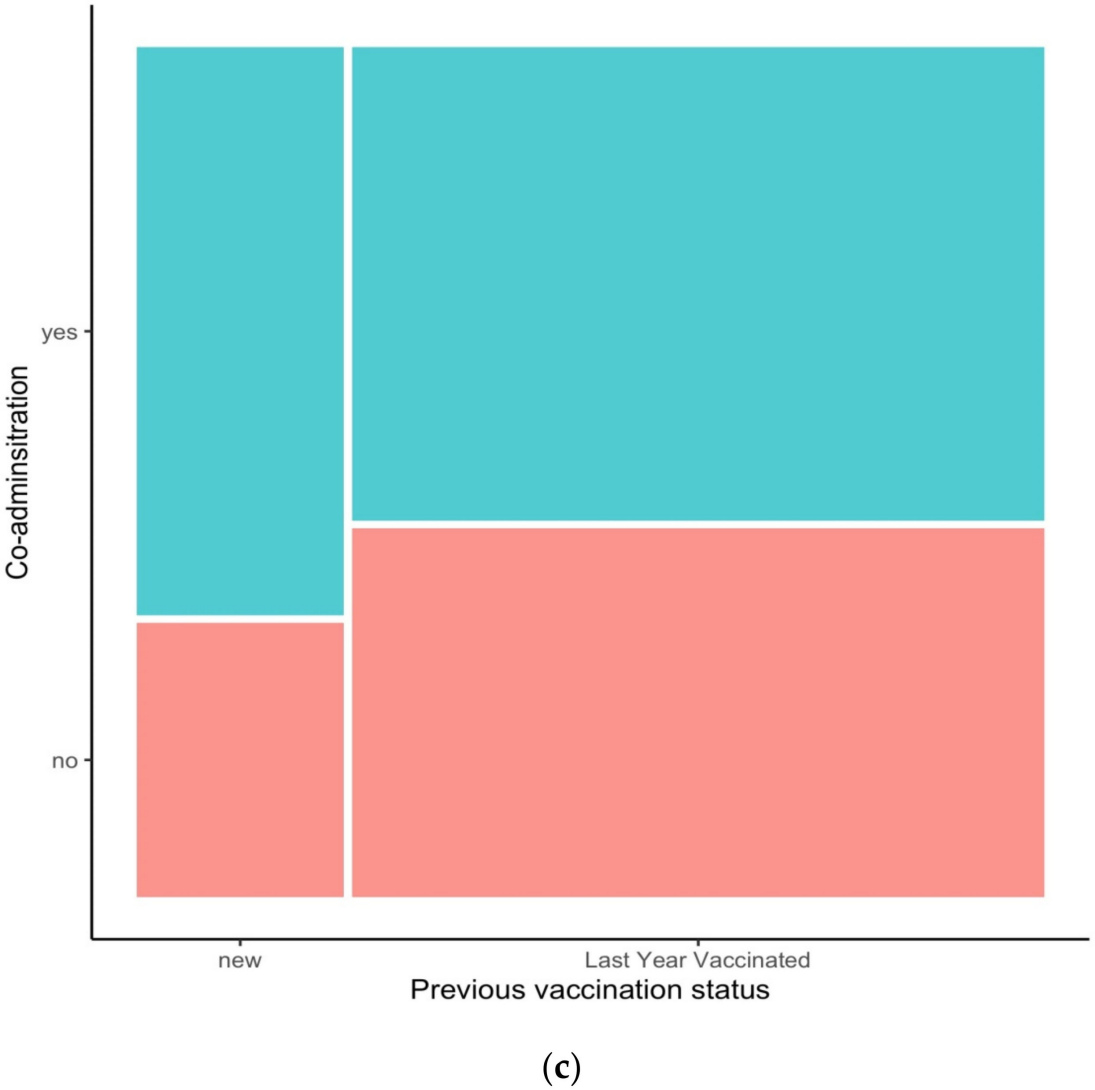
Mosaic plots exploring HCWs’ qualification (**a**), area of activity (**b**), and flu/COVID-19 vaccines coadministration (**c**) in newly vaccinated HCWs (“New”) versus HCWs who got vaccinated also in the 2020–2021 season (“Last Year Vaccinated”).

Figure 2 shows the results of an exploratory analysis conducted by means of mosaic plots on the newly vaccinated HCWs versus HCWs who were also vaccinated in the 2020–2021 season.

Concerning qualification, a possible effect in favor of “new” vaccination is shown by students, residents, professionals belonging to the “Other” group, and auxiliary staff. On the other hand, a behavior consistent with “loyalty” towards influenza vaccination (i.e., those who got vaccinated also in the previous season) can be observed for the administrative staff, nurses, and particularly for physicians. Focusing on the area of activity, HCWs from the Services and the General Medicine areas seem to show a propensity towards being newly vaccinated, in comparison with HCWs from the Administrative and the Specialized Medicine areas, who seem to show a more loyalty-oriented attitude towards influenza vaccination. Interestingly, the third mosaic plot shows that, among the newly vaccinated HCWs, the coadministration of the flu and the COVID-19 vaccine seems to be more prevalent than in HCWs who got vaccinated also in the 2020–2021 season.

**Figure 3:**
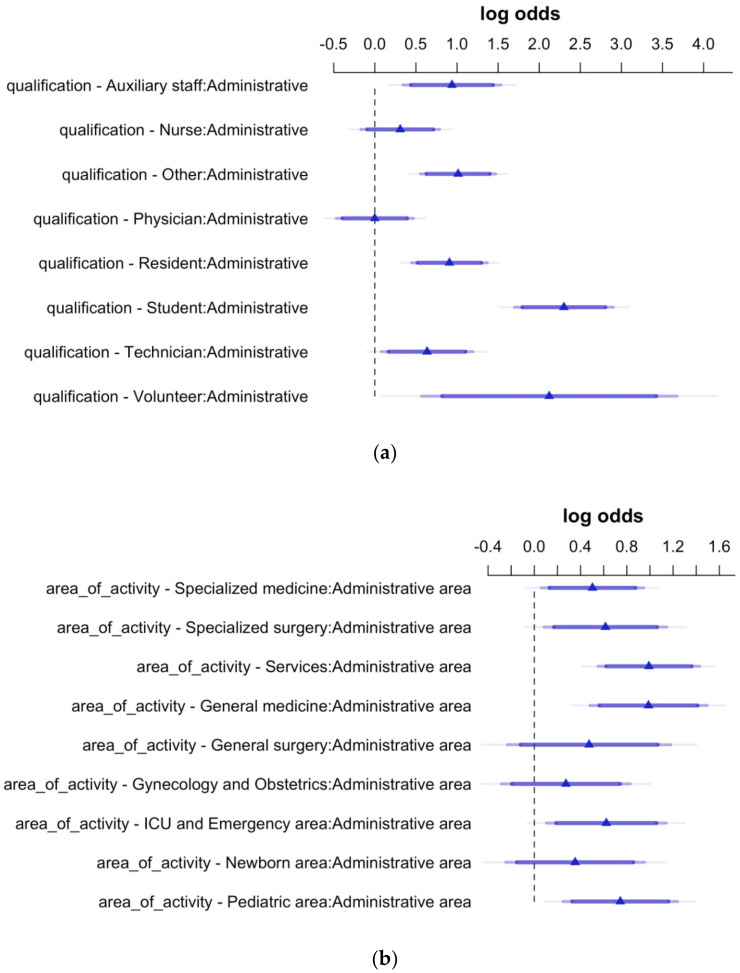
HCWs’ propensity towards being newly vaccinated against influenza-log of the odds and their 95% CIs extracted from two models having HCWs’ qualification (**a**) and area of activity (**b**) as predictor variables. Statistical significance for the effects is shown when the 95% Confidence Intervals do not cross the vertical dashed line set to 0.

In Figure 3, two forest plots show the odds ratios in logarithmic form with their 95% CIs expressing HCWs’ propensity towards being newly vaccinated against influenza, extracted from a model having the variable “qualification” and the variable “area of activity” as a predictor, respectively. The administrative staff and the administrative area were chosen as reference categories as they showed the lowest association towards being newly vaccinated.

Concerning qualification, it can be observed that students, residents, professionals from the “Other” group, and auxiliary staff showed the highest propensity towards being newly vaccinated. Additionally, volunteers are shown to have a meaningful propensity to be newly vaccinated, although this result should be considered with caution due to the scarce numerosity of this group in the total of vaccinated HCWs. Concerning the area of activity, the Services and the General medicine areas were confirmed to have the highest propensity towards being newly vaccinated.

### 3.4. Influenza and COVID-19 Vaccine Coadministration Analysis

Table 4 summarizes frequencies and proportions of HCWs receiving the flu vaccine and/or the COVID-19 vaccine during the campaign, as well as a possible classification based on their attitude towards the vaccine coadministration.

A total of 4165 HCWs were vaccinated with the influenza vaccine and/or the COVID-19 vaccine within the three weeks of the campaign. Out of them, 3526 (84.7%) received the COVID-19 vaccine and 2381 (67.5%) the flu vaccine. Out of the total number of vaccinated HCWs, 1784 (42.8%) chose to receive only the COVID-19 vaccine, therefore were considered “refusers” towards the coadministration of the flu and the COVID-19 vaccine; 342 HCWs (8.2%) chose to receive both the COVID-19 and the flu vaccine within the three weeks of the campaign but on separate days, thus being classified as “intentional delayers”. According to the definition of vaccine hesitancy by WHO [18], these two groups can be collapsed into a single one. As a result, 2126 and 51.0% of vaccinated HCWs in the 2021–2022 campaign were hesitant towards the coadministration of a COVID-19 and a flu vaccine. Additionally, 1400 (33.6%) HCWs decided to receive the COVID-19 and the flu vaccine in the same day by coadministration, showing a “fully compliant” behavior towards WHO’s recommendations on flu and COVID-19 vaccine coadministration [28]. Lastly, 639 (15.3%) HCWs only got the flu vaccination. As, in Italy, COVID-19 vaccination and specifically the booster dose were made mandatory for HCWs by Decree-Law n. 44/2021 and n. 172/2021, respectively [31,32], it is reasonable that HCWs from the latter group had already received the booster dose of a COVID-19 vaccine prior to the start of the campaign, or alternatively could not yet receive it due to timing issues, i.e., 150 days not yet passed from the second dose uptake. As a consequence, they were classified as “unintentional delayers”.

**Figure 4:**
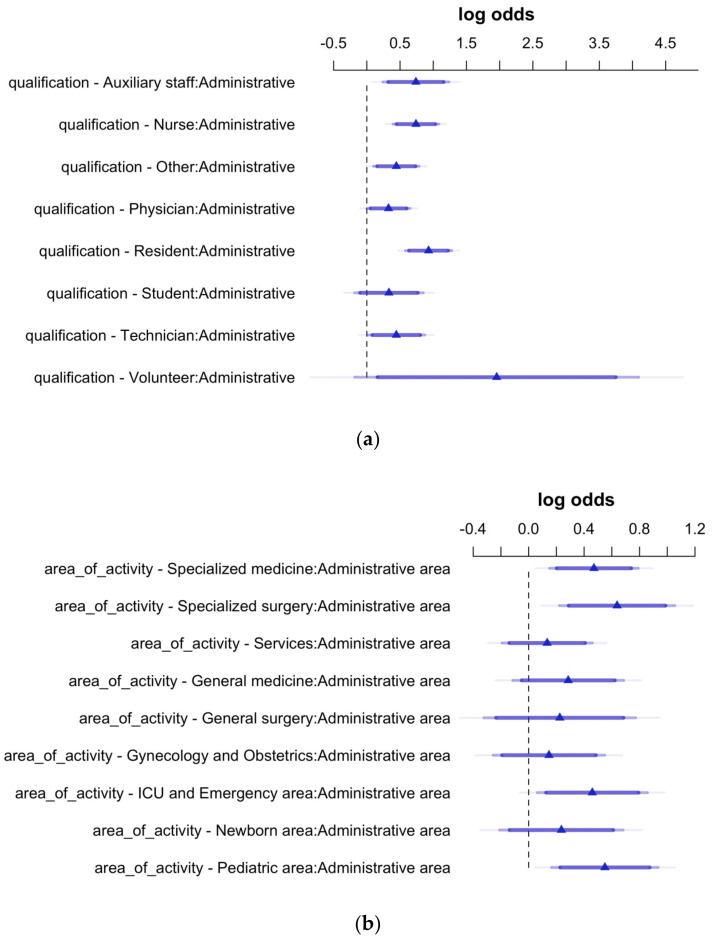
HCWs’ propensity towards influenza and COVID-19 vaccine coadministration-log of the odds and their 95% CIs extracted from two models having HCWs’ qualification (**a**) and area of activity (**b**) as predictor variables. Statistical significance for the effects is shown when the 95% Confidence Intervals do not cross the vertical dashed line set to 0.

In Figure 4, two forest plots show the odds ratios in logarithmic form with their 95% CIs expressing the propensity towards the flu and the COVID-19 vaccine coadministration, related to HCWs’ qualification and area of activity. Again, the administrative staff and the administrative area were chosen as reference categories.

As can be observed, residents showed the highest propensity towards receiving the influenza and the COVID-19 vaccine by coadministration, followed by nurses and the auxiliary staff. Concerning the area of activity, HCWs belonging to the Specialized surgery, Pediatric area, Specialized medicine, and ICU–Emergency are shown to have a significant propensity towards the coadministration of the influenza and the COVID-19 vaccine.

## 4. Discussion

One of the primary evidences of the 2021–2022 influenza vaccination campaign in Fondazione is the achievement of a VCR of 52%. This value considerably increased compared to that of the 2020–2021 campaign, 43.1%, and is far higher than that of the 2019–2020 campaign, 21.5%.

Concerning the fundamental question of which factors could have contributed to this result, it can be noticed that, while a long tradition of hesitancy among HCWs towards flu vaccination has been ascertained even in recent years in Italy, as well as in several European countries [11,12,13,14], with Italian flu VCRs rarely exceeding the threshold of 20%, a meaningful increase was registered in Fondazione in the 2020–2021 and in the last campaign, i.e., in the two years of the COVID-19 pandemic. Similar results have been reported by other Italian [33,34,35] and international studies [36,37,38]. Therefore, it is possible to hypothesize that the SARS-CoV-2 pandemic could have played a significant role in increasing awareness on the importance of vaccination in general and specifically of flu vaccination, thus contributing to increase the flu VCRs, even among HCWs [39,40,41,42,43].

It is worth noticing that the VCR of 52% reached this year is calculated on the total of the internal personnel of the Fondazione. This year, the number of internal personnel was also affected by the portion of new job contracts stipulated with residents, as allowed by Law n. 27/2020, in response to the COVID-19 emergency [44]. Residents in Italy are not usually directly employed by hospitals, but have a scholarship with the University. Due to widespread COVID-19, the need for residents employed with a contract grew to support hospitals during the pandemic. With a contract, residents become considered as internal personnel of Fondazione. On the other hand, the number of students (not a part of the internal personnel of Fondazione) participating in the campaign decreased significantly compared to the two previous campaigns because they were attending online classes while traineeships in the hospital were suspended.

The study evidenced a fluctuation in the median age of HCWs receiving the flu vaccination over the three campaigns. In the 2019–2020 campaign (pre-pandemic), younger HCWs, in particular those aged 18–39 years, were the most represented among the vaccinated population, setting the median age at 36. This result is consistent with other Italian studies [33]. In the 2020–2021 campaign, the first contemporary to the pandemic, the median age of the vaccinated population increased to 43. This result reflects the fact that, during the first year of the COVID-19 pandemic, a dramatic increase in vaccinated HCWs aged 40–59 years occurred. Again, a similar observation was reported in other Italian contexts [33]. Indeed, we can assume that the effect of widespread SARS-CoV-2 pushed older HCWs to consider influenza vaccination because, as shown in some research, it seems to reduce both the incidence of infection as well as the severity of COVID-19 [45]. Moreover, older adults, being at higher risk for COVID-19, were strongly invited to get protection against influenza in order to avoid confusion and facilitate the differential diagnosis between influenza and COVID-19 [46]. Finally, the important decrease in the median age observed in the 2021–2022 campaign is caused by the fact that HCWs aged 18–39 years returned to represent by far the largest group among vaccinated HCWs, registering a steep increase, while HCWs aged 40–59 years remained substantially stable. We found difficulties in assessing whether a similar result was observed in other contexts. In summary, it can be speculated that, whilst in the 2020–2021 campaign the increased adherence towards flu vaccination was substantially sustained by older HCWs, i.e., the age group most affected by potential complications from COVID-19 and therefore most sensitized towards the need for vaccination against respiratory infections, first of which influenza, in the 2021–2022 campaign younger HCWs recuperated, returning to be the most represented group among the vaccinated.

Proceeding with the analysis, the most represented professional categories within the vaccinated population were physicians, residents, and nurses. Whilst physicians have long been considered one of the most adherent professional categories towards flu vaccination [47], even without reaching the optimal coverage target [48], results from residents and nurses might be considered more surprising. Particularly, despite a long tradition of vaccine hesitancy found in the scientific literature for nurses [11,49], this professional group nearly tripled among the vaccinated population in the 2020–2021 campaign [25]. Concerning residents, our study showed their leading role in the 2021–2022 campaign, as the number of those who received flu vaccination nearly doubled. This is in contrast with a possible hesitant behavior towards flu vaccination by this professional group shown in literacy, mainly due to low perceived risk of either contracting flu or developing severe infection [21,50].

Thanks to the questionnaire, it was possible to investigate the reasons that prompted HCWs to get vaccinated. Reason 1, i.e., “Vaccination is the most effective strategy of prevention” was the most popular, reaching 81%, followed in second place by reason 9, i.e., “As HCW it’s my duty to get vaccinated to protect my patients”. Ultimately, the group of COVID-19-related reasons ranked third as a preference of choice.

Whilst the choice of reason 1 is not that informative, as it was expressed by the vast majority of HCWs independently from age, professional category, and area of activity, and also it is the most “general” motivation behind any flu vaccination program, interesting considerations could be made for reason 9 and for the COVID-19-related group of reasons. Reason 9, which expressed the choice of vaccination to protect patients, has been significantly associated with residents. Again, this might be viewed in contrast with the traditional young HCW’s “profile”, whose attitude towards flu vaccination may be driven by self-utility factors, such as self-protection, in case of vaccine acceptance, or low perceived risk of threat for oneself, in case of refusal [51,52].

Conversely, COVID-19-related reasons were chosen by HCWs showing a consistently higher median age than that of the general vaccinated population, suggesting that COVID-19 has had a substantial effect on the involvement of older adults in the first place. Additionally, it is noteworthy that this group of reasons was significantly associated with the administrative staff, revealing how COVID-19 has come to sensitize even groups of health workers not in direct contact with patients and/or not directly employed in hospital wards.

The newly vaccinated rate in the current campaign decreased compared to the 2020–2021 season (23.3% vs. 40.2%). This fact, however, must be interpreted in the light of the unprecedented increase of the newly vaccinated subgroup occurred in the 2020–2021 campaign [25]. The categories of residents, students, auxiliary staff, and professionals belonging to the “Other” group were more prone to be newly vaccinated, i.e., having received flu vaccination in the current campaign but not in the previous one. In particular for residents and the “Other” group, these findings are consistent with the steep increase in absolute numbers of vaccinated individuals belonging to these two categories. As already mentioned, residents confirmed to have played a leading role in the present campaign, for their renewed and noticeable adherence to flu vaccination, compared to the previous one. To date, we have not found evidence from recent literature regarding a similar phenomenon in other Italian or international contexts. However, some previous studies already reported residents reaching flu immunization rates higher than the national average for HCWs [53], especially for certain specialties such as Infectious Diseases, Pediatrics, Hematology, and Hygiene [54]. Moreover, other studies suggested that residents might be a professional category in which to invest educational and training efforts in order to improve vaccine acceptance of future physicians [55]. Concerning the “Other” category, this included a wide heterogeneity of professionals such as biologists, physicists, pharmacists, psychologists, and engineers. These findings again confirmed that, in the COVID-19 pandemic era, a wider flu vaccine acceptance, even among HCWs who do not work in clinical wards, seemed to occur. Moreover, as these professionals are usually employed in laboratory, radiology, pathology, and other hospital supporting functions, this explains the significant association between the “Services” area and the propensity to be newly vaccinated.

A remarkable aspect is that the flu/COVID-19 vaccine coadministration was more prevalent among the newly vaccinated group. Although this observation came from an explorative analysis (mosaic plot), we believe it may deserve some considerations. Indeed, it can be speculated that the contemporary offer of the two vaccines might act as a facilitator towards the flu vaccine uptake for HCWs who accessed the vaccination service this time but did not get the flu vaccine in the 2020–2021 season. In other words, the possibility of coadministration might have been successful in also reaching those HCWs who were otherwise hesitant towards the flu vaccine alone. Similarly, an encouraging acceptance of the flu and the COVID-19 co-vaccination in the 2021–2022 campaign was also observed, though results are preliminary, by other Italian authors [56].

According to the WHO’s definition of vaccine hesitancy [18], 51% of Fondazione’s HCWs can be considered hesitant towards the flu/COVID-19 vaccine coadministration. This means they entirely refused the flu vaccine administration or differed it from the COVID-19 shot, although coadministration was available and recommended [28,29]. Considering that this reluctance is shown by a professional category informed and sensitized about health and prevention issues, these data may be surprising and even worrying, given the influence that HCWs attitudes and behaviors might exert on the general public [57]. However, a long tradition of vaccine hesitancy among HCWs has already been discussed in the present study and in the scientific literature [9,21], proving that a lot of work remains. In particular, the administrative staff was shown to be the major refuser of co-vaccination. Interestingly, the most expressed justification collected on the field by our vaccinating teams was a purely non-sanitary motivation, represented by the belief that coadministration could be more dangerous and problematic than the single administration. This concern is not new among HCWs and was already reported in the literature [58]. However, this finding points out the consistent need for educational interventions even among HCWs, with the aim to debunk the most common misconceptions on vaccination, including those related to simultaneous vaccine administration, in order to increasingly improve immunization acceptance [59].

On the other hand, 33.6% of HCWs were fully compliant towards vaccine coadministration. Residents, followed by nurses, showed the highest propensity to receive both vaccinations. These data may be compared with another Italian study showing a meaningful acceptance of the flu/COVID-19 vaccine coadministration among HCWs, with the highest adherence observed among residents and physicians, whereas a certain reluctance was registered among nurses [56].

Following the results of our study and the related discussion, it is now of utmost importance to draw general considerations on which interventions should also be confirmed for the 2022–2023 influenza vaccination campaign at Fondazione, which ones should be improved, and which ones should be implemented for the first time.

As discussed above, an educational communication campaign is indispensable to consistently inform HCWs decisions on flu and/or COVID-19 vaccination [59], helping to confute old (fear of vaccine adverse events or skepticism on vaccine effectiveness and/or disease severity [9,21]) and renewed (fear of adverse events related to flu/COVID-19 co-vaccination [56,58]) misconceptions. The analysis of the most expressed reasons behind flu vaccine acceptance allowed us to realize that at least two core messages should be conveyed to promote and consolidate immunization rates in future campaigns, and that different professional subgroups would differently respond to those messages: (i) the need to be vaccinated in order to protect patients—an “altruistic” purpose shared in particular by younger HCWs, first of all residents, and all HCWs employed in a clinical context [60]; and (ii) the importance of getting vaccinated to seek self-protection, a message that would convince especially older HCWs and/or those not in direct contact with patients.

Concerning the gaming strategy, it is difficult to draw conclusions on its effectiveness. Although this intervention has proven to be effective in evidence-based literature and is suggested by WHO [61,62], feedback from the field revealed that a significant portion of HCWs were unaware of a competition among hospital departments, due to infrequent access to the hospital intranet. Correlated to this consideration, given that both the educational/promotional intervention and the gaming strategy have been implemented mainly through this online platform, we believe that an enlargement of the communication scope is needed for the next campaign, e.g., through an opening ceremony or a press conference attended by the hospital management and ward directors, in an effort to better publicize the event [17,61].

Concerning the organization of vaccine delivery, the revised logistical model based mainly on a central large hub does not appear to have negatively impacted the flu vaccine uptake, given the consistent improvement of the hospital VCR. Moreover, we believe this model should also be adopted in the 2022–2023 flu vaccination campaign, as it made the coadministration of the flu and the COVID-19 vaccine technically feasible, an element which proved to be a promising leverage to consolidate/improve flu immunization rates [56].

In a recent review by Schumacher et al., the authors concluded that strategies based on education and promotion or on-site vaccination are effective in obtaining a relative increase in flu immunization rates, although these rarely exceed the value of 40%. On the contrary, harder interventions such as mandatory vaccination policies or mandatory declination proved effective in reaching flu vaccination coverage in HCWs of over 90% [63]. Although a hard debate on mandatory flu vaccination for HCWs is long-standing and still in place [64,65], the extremely positive results obtained through mandatory COVID-19 vaccination for HCWs in Italy, both in terms of immunization rates (>98%) [66] and prevented infections [67,68,69], allow us to conclude that influenza vaccination coverage among HCWs would undoubtedly benefit from this policy.

This study has some limitations. Firstly, we focused on investigating the reasons behind HCWs’ decision to get vaccinated against flu. As mentioned, this approach could be greatly helpful in order to understand the main drivers towards flu vaccination acceptance, allowing us to conceive tailored strategies that would stress and/or reinforce those drivers, e.g., a communication campaign highlighting the ethical obligation for HCWs to get vaccinated to protect their patients, or recalling the threat posed by respiratory infections, primarily of SARS-CoV-2, thus the need to vaccinate to avoid them. However, an investigation into the reasons for not getting vaccinated could be equally helpful and informative, and this was not performed in this campaign. Secondly, in our analysis, we did not investigate a possible role of a previous positivity to SARS-CoV-2 in relation to a potential hesitancy towards flu vaccination, a phenomenon observed by other authors and shown to be a relevant determinant of vaccine hesitation [30]. This is to be included as a potential item of analysis in the next vaccination campaign by introducing a specific question in the questionnaire. Finally, another limitation in the current study is the lack of formal tools to assess the effectiveness of the implemented strategies: a consistent improvement of the flu immunization rates has been clearly highlighted, however it is hard to assess the qualitative and quantitative contribution of each single strategy to these results. This limitation is urgently to be overcome in the next vaccination campaign by implementing ad hoc analysis.

On the other hand, an important point of strength of the present study resides in the continuous observation of Fondazione’s HCWs for three consecutive seasons, thus contributing to characterize HCWs’ attitude towards flu vaccination and its possible evolution in the COVID-19 era, in order to better inform influenza vaccination program decision makers.

## 5. Conclusions

In the 2021–2022 influenza vaccination campaign for HCWs in Fondazione, a VCR > 50% (52.0%) was reached for the first time, confirming the positive trend started in the 2020–2021 campaign [25]. A great contribution by the COVID-19 pandemic in renewing HCWs’ awareness towards the need for protection against respiratory infections, primarily influenza, is highly probable and widely highlighted in the literature [33,34,35,36,37,38,39,40,41,42,43].

Whilst the increase of flu VCR in the 2020–2021 season was sustained mainly by HCWs aged 40–59 years, in the present campaign those aged 18–39 years recuperated, returning to be the most represented in the vaccinated population.

Consistently, whilst physicians continued to be the most loyal professional category towards flu vaccination, residents were one of the leading actors of the present campaign, doubling their numbers, as demonstrated by their quota among the newly vaccinated HCWs. This was also observed in other preliminary studies [56], however, recent literature concerning residents’ attitude towards flu vaccination is scarce, and further research is urged to better understand barriers and motivators behind their intention to get vaccinated, given that they would represent the future medical class [55].

An evolution might have occurred even in HCWs’ reasons for seeking flu vaccination, as an “altruistic” reason, i.e., “As HCW it’s my duty to get vaccinated to protect my patients”, was the second most expressed, again associated with residents; this is partially in contrast with past literature showing that self-protection is often a major determinant for HCWs intention to get the flu vaccine [9,21,70]. Furthermore, COVID-19-related reasons ranked as the third group of reasons expressed by vaccinated HCWs, more associated with older HCWs (particularly to the administrative staff).

The 2021–2022 campaign was the first in which HCWs were offered the possibility of undergoing simultaneous vaccination against both influenza and COVID-19. Interestingly, coadministration was utilized more frequently by the newly vaccinated group, suggesting that it might be a valuable tool to intercept HCWs previously hesitant towards the flu vaccination alone [56]. However, hesitancy towards COVID-19 and towards flu vaccination might influence each other [30], as well as a further component of vaccine hesitancy towards co-vaccination, even in HCWs, might be a long-standing misconception about additional side/adverse effects in comparison to receiving the two vaccinations separately [58]. Indeed, a large proportion of hesitant HCWs towards co-vaccination was observed in our study (51.0%). Interestingly, among the 33.6% HCWs fully compliant with WHO and Italian health authorities recommendations on co-vaccination [28,29], residents were again distinguished. As an influenza/COVID-19 co-vaccination might be, at least in the very next years, an irrevocable tool to ensure population protection against what was called a “twindemic” for mankind [71], future research should promptly address HCWs’ attitude towards this practice.

Concerning future strategies to be implemented in influenza vaccination programs, we believe, also in light of the findings of our study, that a solid communication, before and during the flu vaccination campaign, should always be utilized. This should be tailored in order to stimulate different motivations in different HCWs subgroups, e.g., the duty to protect patients when focusing on younger HCWs, or recalling the threat posed by respiratory infections when focusing on older ones. In addition this should constantly address and debunk old and new misconceptions regarding flu vaccination and/or flu/COVID co-vaccination [59]. As co-vaccination emerged as a promising tool to consolidate flu immunization rates [56], this should be firmly encouraged and persuaded, also through optimal logistical organization. Finally, given the unquestionable results, in terms of immunization rates and the mitigation of COVID-19 burden, achieved by mandatory COVID-19 vaccination for HCWs in Italy [66,67,68,69], also mandating influenza vaccination [63] would be another powerful if not decisive arrow in our quiver.

## Figures and Tables

**Table 1:** Features of vaccinated HCWs; season 2021–2022, 2020–2021, and 2019–2020.

Healthcare Workers (HCWs)	2021–2022		2020–2021		2019–2020	
Internal personnel vaccinated (n)	1795		1816		733	
Students, residents, outsourc. personnel vaccinated (n)	586		287		420	
Total personnel vaccinated (n)	2381		2103		1153	
Overall hospital internal personnel (n)	3451		4213		3405	
Vaccination Coverage Rate (VCR) (%)	52.0		43.1		21.5	
Gender (n, %)						
F	1584	66.5	1418	67.5	740	64.2
M	797	33.5	685	32.5	413	35.8
Age						
Median; IQR	39; 23		43; 23		36; 25	
18–39 (n, %)	1204	50.6	938	44.6	642	55.7
40–59 (n, %)	916	38.5	925	44	374	32.4
>60 (n, %)	261	11.0	240	11.4	137	11.9
Qualification (n, %)						
Physician	603	25.3	600	28.5	283	24.5
Resident	416	17.5	219	10.4	238	20.6
Nurse	414	17.4	452	21.5	171	14.8
Student	83	3.5	158	7.5	166	14.4
Technician	151	6.3	155	7.4	96	8.3
Auxiliary staff	100	4.2	120	5.7	34	2.9
Administrative	196	8.2	196	9.3	48	4.2
Volunteer	7	0.3	0	0.0	16	1.4
Other	409	17.2	203	9.7	100	8.7
NA	2	0.1	0	0.0	1	0.1
Area of activity (n, %)						
General medicine	181	7.6	186	8.8	226	19.6
Specialized medicine	554	23.3	716	34	374	32.4
ICU and Emergency	193	8.1	183	8.7	72	6.2
General surgery	69	2.9	37	1.8	44	3.8
Specialized surgery	172	7.2	286	13.6	170	14.7
Gynecology—Obstetrics *	174	7.3	-	-	-	-
Pediatric area	224	9.4	245	11.7	117	10.1
Newborn area	125	5.2	173	8.2	97	8.4
Administrative	212	8.9	275	13.1	27	2.3
Services *	468	19.7	-	-	-	-
Other	0	0.0	0	0.0	15	1.3
NA	9	0.4	2	0.1	11	1.0

* Categories introduced for the first time in the 2021–2022 campaign.

**Table 2:** Reasons for influenza vaccination uptake; season 2021–2022. Reasons are progressively listed as they were displayed in the questionnaire. Note that, as every HCW could express more than one reason (maximum two), cumulative sum of relative frequencies for each reason overcomes 100%.

	Reason 1		Reason 2		Reason 3		Reason 4		Reason 5		Reason 6		Reason 7		Reason 8		Reason 9	
	“Vaccination Is the Most Effective Strategy of Prevention”		“COVID-19 Pandemic Made Me Aware That Vaccination Is Useful for Myself”		“COVID-19 Pandemic Made Me Aware That Vaccination Is an Act of Responsibility towards the Community”		“COVID-19 Pandemic Made Me Aware of the Danger of Respiratory Infections”		“As HCW I Am More Exposed to the Flu”		“I Fear the Complications of the Flu”		“I Suffer from Diseases at Risk of Complications”		“I Live with Fragile Patients”		“As HCW It’s My Duty to Get Vaccinated to Protect My Patients”	
n, %	1928	81.0	197	8.3	330	13.9	59	2.5	373	15.7	77	3.2	82	3.4	174	7.3	766	32.2
Gender (n, %)																		
F	1250	78.9	123	7.8	249	15.7	42	2.7	243	15.3	51	3.2	59	3.7	132	8.3	512	32.3
M	677	84.9	74	9.3	81	10.2	17	2.1	130	16.3	26	3.3	23	2.9	42	5.3	254	31.9
Age																		
median; IQR	39; 23		44; 22		40; 22		46; 23.5		36; 22		47; 22		53.5; 13		45.5; 21		33; 18	
18–39 (n, %)	997	82.8	88	7.3	160	13.3	22	1.8	213	17.7	29	2.4	14	1.2	69	5.7	505	41.9
40–59 (n, %)	717	78.3	88	9.6	142	15.5	27	2.9	119	13.0	36	3.9	46	5.0	89	9.7	214	23.4
>60 (n, %)	214	82.0	109	41.8	28	10.7	10	3.8	41	15.7	12	4.6	22	8.4	16	6.1	47	18.0
Qualification (n, %)																		
Physician	496	82.3	46	7.6	66	10.9	11	1.8	112	18.6	21	3.5	19	3.2	36	6.0	229	38.0
Resident	355	85.3	15	3.6	37	8.9	6	1.4	84	20.2	6	1.4	2	0.5	14	3.4	223	53.6
Nurse	323	78.0	28	6.8	53	12.8	11	2.7	60	14.5	9	2.2	25	6.0	44	10.6	155	37.4
Student	67	80.7	11	13.3	11	13.3	4	4.8	16	19.3	1	1.2	0	0.0	7	8.4	33	39.8
Technician	118	78.1	14	9.3	23	15.2	4	2.6	19	12.6	8	5.3	9	6.0	19	12.6	36	23.8
Auxiliary staff	68	68.0	5	5.0	16	16.0	0	0.0	22	22.0	5	5.0	5	5.0	10	10.0	23	23.0
Administrative	153	78.1	34	17.3	57	29.1	8	4.1	16	8.2	6	3.1	7	3.6	20	10.2	10	5.1
Volunteer	5	71.4	0	0.0	0	0.0	1	14.3	1	14.3	0	0.0	0	0.0	1	14.3	2	28.6
Other	342	83.6	44	10.8	67	16.4	13	3.2	42	10.3	21	5.1	14	3.4	23	5.6	55	13.4
NA	1	50.0	0	0.0	0	0.0	1	50.0	1	50.0	0	0.0	1	50.0	0	0.0	0	0.0
Area of activity (n, %)																		
General medicine	150	82.9	16	8.8	18	9.9	5	2.8	37	20.4	5	2.8	3	1.7	12	6.6	70	38.7
Specialized medicine	445	80.3	44	7.9	63	11.4	13	2.3	99	17.9	11	2.0	18	3.2	27	4.9	247	44.6
ICU and Emergency	159	82.4	15	7.8	22	11.4	6	3.1	43	22.3	7	3.6	3	1.6	16	8.3	68	35.2
General surgery	63	91.3	6	8.7	10	14.5	0	0.0	18	26.1	0	0.0	3	4.3	4	5.8	16	23.2
Specialized surgery	135	78.5	9	5.2	17	9.9	4	2.3	23	13.4	2	1.2	7	4.1	18	10.5	73	42.4
Gynecology—Obst.	136	78.2	9	5.2	28	16.1	5	2.9	35	20.1	8	4.6	7	4.0	9	5.2	61	35.1
Pediatric area	178	79.5	11	4.9	21	9.4	3	1.3	36	16.1	7	3.1	8	3.6	16	7.1	105	46.9
Newborn area	103	82.4	14	11.2	19	15.2	1	0.8	15	12.0	4	3.2	1	0.8	9	7.2	56	44.8
Administrative	161	75.9	30	14.2	56	26.4	9	4.2	14	6.6	13	6.1	10	4.7	20	9.4	12	5.7
Services	391	83.5	42	9.0	75	16.0	13	2.8	51	10.9	20	4.3	22	4.7	43	9.2	56	12.0
Other	0	0.0	0	0.0	0	0.0	0	0.0	0	0.0	0	0.0	0	0.0	0	0.0	0	0.0
NA	7	77.8	1	11.1	1	11.1	0	0.0	2	22.2	0	0.0	0	0.0	0	0.0	2	22.2

**Table 3:** HCWs’ previous influenza vaccination status; season 2021–2022, 2020–2021, and 2019–2020.

HCWs	2021–2022		2020–2021		2019–2020	
Total vaccinated (n)	2381		2103		1153	
Previous vaccination (n, %)	§		#		#	
yes	1834	76.7	1258	59.8	777	67.4
no	547	23.3	845	40.2	376	32.6

§ In season 2021–2022 HCWs were asked if they received the flu vaccination also in the previous season (2020–2021); # In season 2020–2021 and 2019–2020 HCWs were asked if they ever received the flu vaccination before.

**Table 4:** HCWs’ influenza vaccine and/or COVID-19 vaccine uptake; attitude towards vaccine coadministration.

Vaccine Uptake	n	%	Attitude
Total vaccine uptake	4165		
Total COVID-19 vaccine uptake	3526	84.7	
Total Flu vaccine uptake	2381	67.5	
**Influenza AND COVID-19 vaccine uptake**			
COVID-19 + Flu vaccine by co-admin.	1400	33.6	Fully compliant
COVID-19 + Flu vaccine separated	342	8.2	Intentional delayers
**Influenza OR COVID-19 vaccine uptake**			
Flu vaccine only	639	15.3	Unintentional delayers
COVID-19 vaccine only	1784	42.8	Refusers

## Data Availability

Data reported in Section 3 were retrieved from a database in which anonymous questionnaires and anonymized COVID-19 vaccination data were entered. This database is in the availability of the Director of the Postgraduate School of Public Health, University of Milan.
